# Inside information on xenon adsorption in porous organic cages by NMR†
†Electronic supplementary information (ESI) available: Additional details of experimental methods and results as well as computational modelling. See DOI: 10.1039/c7sc01990d



**DOI:** 10.1039/c7sc01990d

**Published:** 2017-06-14

**Authors:** Sanna Komulainen, Juho Roukala, Vladimir V. Zhivonitko, Muhammad Asadullah Javed, Linjiang Chen, Daniel Holden, Tom Hasell, Andrew Cooper, Perttu Lantto, Ville-Veikko Telkki

**Affiliations:** a NMR Research Unit , University of Oulu , P.O.Box 3000 , 90014 Oulu , Finland . Email: ville-veikko.telkki@oulu.fi; b Laboratory of Magnetic Resonance Microimaging , International Tomography Center SB RAS , Department of Natural Sciences , Novosibirsk State University , Instututskaya St. 3A, Pirogova St. 2 , 630090 Novosibirsk , Russia; c Department of Chemistry , Centre for Materials Discovery , University of Liverpool , Crown Street , Liverpool L69 7ZD , UK

## Abstract

A solid porous molecular crystal formed from an organic cage, CC3, has unprecedented performance for the separation of rare gases. Here, xenon was used as an internal reporter providing extraordinarily versatile information about the gas adsorption phenomena in the cage and window cavities of the material. ^129^Xe NMR measurements combined with state-of-the-art quantum chemical calculations allowed the determination of the occupancies of the cavities, binding constants, thermodynamic parameters as well as the exchange rates of Xe between the cavities. Chemical exchange saturation transfer (CEST) experiments revealed a minor window cavity site with a significantly lower exchange rate than other sites. Diffusion measurements showed significantly reduced mobility of xenon with loading. ^129^Xe spectra also revealed that the cage cavity sites are preferred at lower loading levels, due to more favourable binding, whereas window sites come to dominate closer to saturation because of their greater prevalence.

## Abstract 2

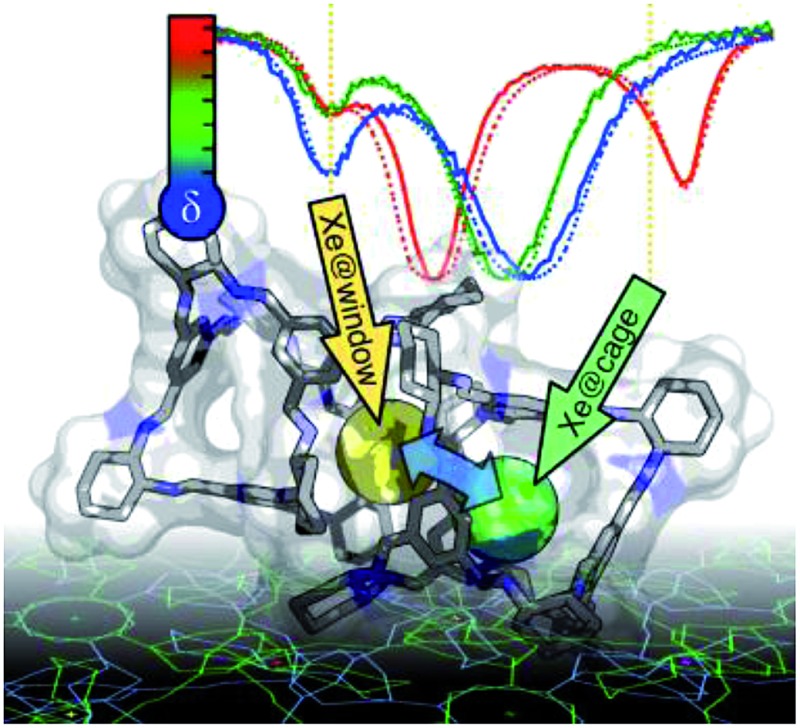
In-depth experimental and computational ^129^Xe NMR analysis of extraordinarily efficient adsorption of xenon in a porous organic cage.

## Introduction

Porous materials are ubiquitous and they have a wide range of important applications, including molecular separations and catalysis.^1^ They provide an alternative means to capture greenhouse gases, such as CO_2_ and CH_4_, as well as valuable noble gases (Xe, Ar, Kr), being potentially more energy efficient than traditional cryogenic methods. Zeolites,^2^ metal–organic frameworks (MOFs),^3^ covalent organic frameworks (COFs)^4^ and porous polymers^5^ have been studied intensively for selective isolation of a certain component in a gas mixture. So far, none of them has supplanted zeolites, although each has their own strengths in specific cases.

Xenon is widely used in optics and medical applications, and it plays important role in nuclear fission processes.^6^ However, its extraction from air is difficult because of the low abundance in the atmosphere (0.087 ppm by volume)^6*b*^ and its inert nature, leading to a high commercial price. Because of its inertness, the selective isolation of xenon by porous materials requires tight size selectivity.^7^ Additionally, the adsorbent should have a high adsorption capacity for commercial utilization.

**Fig. 1:**
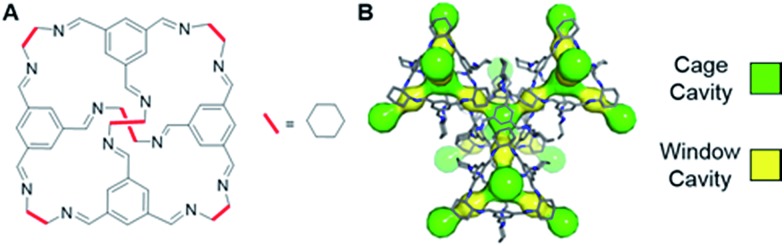
(A) Chemical structure of CC3 cage. (B) 3D crystal and cavity structure of CC3 material. The cage and window cavities are illustrated by green and yellow, respectively.

Recently, it was reported that an organic cage molecule, CC3,^8^ has unprecedented performance in the solid state for the separation of rare gases.^9^ This selectivity arises from precise size match between the rare gas and the organic cage. Separation of krypton, xenon and argon from air at concentrations of only a few parts per million has become feasible. The tetrahedral CC3 cage is structured by imine bonds that connect rigid aromatic rings to the more flexible cyclohexane linkers (Fig. 1A). The cage molecule packs into a crystalline structure forming an interconnected 3D pore structure *via* cage windows (Fig. 1B). The molecular framework has the largest inclusion sphere of 4.4 Å in the cage cavity, which is close to the diameter of xenon (4.10 Å) and other higher-mass noble gases. The narrowest point in the pore channels between the cage and window cavities is only 3.6 Å in diameter; that is, smaller than xenon. However, the vibrational motion of the cage molecules allows the movement of xenon between the cavities. Even SF_6_ with the kinetic diameter of 5.5 Å can enter the cage because of thermal motion and flexibility of these organic crystals.^10^

Xenon has a ^129^Xe isotope with spin-1/2 as well as relatively high natural abundance (26%) and NMR receptivity. The chemical shift of ^129^Xe is extremely sensitive to its local environment, and its nuclear spin polarization can be increased by several orders of magnitude by spin-exchange optical pumping (SEOP). Therefore, it is an excellent inert probe molecule in NMR and magnetic resonance imaging (MRI) applications in chemistry, biochemistry, materials science and medicine.^11^ It has been exploited, for example, in MRI of lungs,^12^ microfluidic flow imaging,^13^ investigation of liquid crystals,^14^ polymers,^15^ and ionic liquids,^16^ as well as the determination of pore sizes of porous media.^17^ Xenon trapped inside of a cage molecule (typically a cryptophane cage), which is functionalized to bind to a specific target, is also used as an NMR biosensor. Combination of hyperpolarization and chemical exchange saturation transfer (CEST)^18^ techniques enables very high-sensitivity, background-free molecular imaging.^19^ Finding alternative, optimized and lower cost cages for xenon biosensor applications is also an important branch of research.^20^ Hyperpolarized CEST technique has also been exploited in materials research, and it enables the observation of Xe-binding sites that are otherwise invisible by direct detection methods in, *e.g.*, spores, proteins, cryptophane and cucurbituril.^21^

Here, we use xenon, *via* its rich chemical and dynamical ^129^Xe NMR information, as an internal probe for adsorption phenomena in CC3. We show that the combination of various experimental ^129^Xe NMR techniques with the state-of-the-art quantum chemical calculations provide exceptionally versatile information about binding, occupancies, dynamics and equilibrium of xenon in CC3.

## Results and discussion

### Samples

Three homochiral CC3-R^9^ samples with different xenon loadings were prepared. The samples contained approximately 4 cm of solid CC3-R powder and Xe gas in a sealed 5 mm sample tube (see details in the ESI†). The Xe : CC3-R molar ratio for the low loading (LL), middle loading (ML) and high loading (HL) samples were 0.10 : 1, 0.52 : 1 and 2.4 : 1, respectively. The last sample represents an almost fully saturated material, in which nearly all three binding sites (one in each cage cavity plus four more shared between two cages in the surrounding window cavities) are occupied by xenon.

### ^129^Xe NMR spectra

**Fig. 2:**
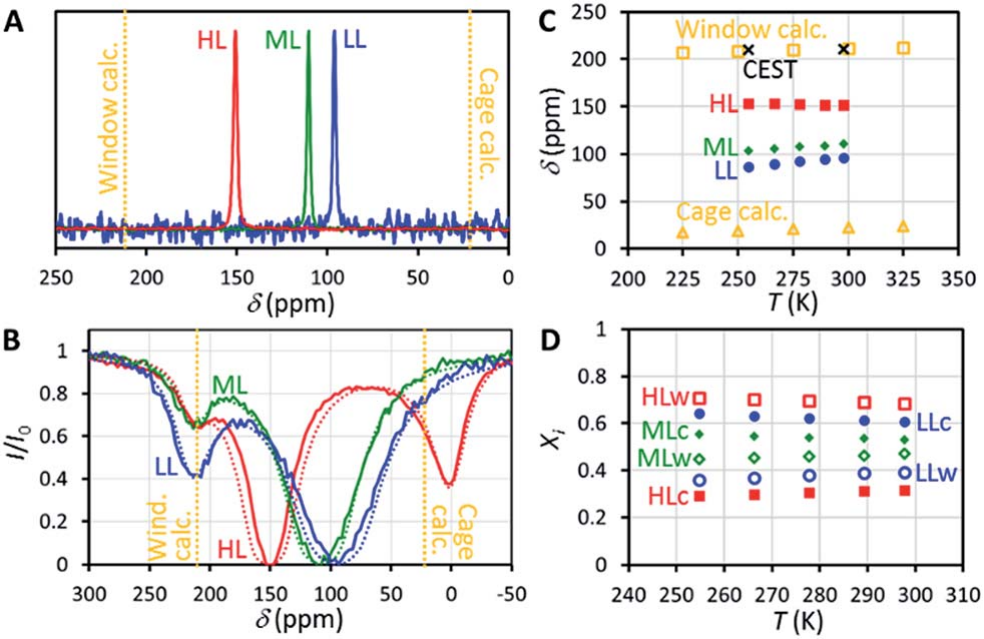
(A) ^129^Xe NMR spectra of xenon adsorbed in CC3-R measured at room temperature. The samples with low, middle and high xenon loading are labelled by LL, ML and HL, respectively. (B) ^129^Xe experimental (solid line) and simulated (dashed line) room temperature CEST spectra. For the HL sample, the length of the saturation pulse was 10 s and *B*_1_ was 5.7 μT. Corresponding values for the LL and ML samples were 5 s and 31 μT. (C) Experimental and calculated chemical shifts. (D) Relative populations of xenon atoms in the cage and window cavities, estimated from the chemical shifts.

^129^Xe NMR spectra of the samples measured at 14.1 T (resonance frequency of ^129^Xe is 166 MHz) at room temperature, shown in Fig. 2A, include a single, relatively narrow (linewidth 1–2 ppm) and symmetrical peak, indicating that the chemical exchange between the cage and window cavities is fast in NMR time scale.^22^ Even below 160 K, the linewidth is so narrow (about 10 ppm) that the system is clearly in the fast exchange region (see Fig. S2 in the ESI†). No signal of free gas around 0 ppm is visible because of the exceptionally high adsorption affinity of xenon in CC3-R.

Chemical shift of ^129^Xe in CC3-R increases with loading (see Fig. 2C). For the HL sample, the shift is almost constant (slope –0.045 ppm K^–1^) over the whole measurement temperature region (255–298 K), while for the lower loading samples it increases with temperature, with the slopes of +0.160 and +0.219 ppm K^–1^ for the ML and LL samples, respectively.

### Quantum chemical calculations

In order to understand the experimental observations, we performed quantum chemical density functional theory (DFT) calculations of the chemical shift of xenon (with respect to atomic Xe) inside static model structures of the cage and window cavities (see Fig. S17†). Nonrelativistic (NR) potential energy and NMR shielding calculations were performed using the dispersion-corrected (D3)^23^ hybrid BHandHLYP^24^ functional, which has been demonstrated to provide the best estimation for NR shielding contribution to both heavy element chemical shifts in molecules^25^ and for Xe chemical shift inside cavities.^20,26^ DFT potential energy and NMR shielding calculations were performed with the Turbomole^27^ code, whereas the Amsterdam Density Functional^28^ program package was used for relativistic calculations of the Xe NMR shielding at the zeroth-order regular approximation level of theory including scalar (SR-ZORA) or both scalar and spin–orbit (SO) relativistic effects (SO-ZORA)^28^ (see details in the ESI†). All-electron co-r^20,29^/def2-SVP^30^ (NR) and jpcl/TZP^31^ (ZORA) basis sets were used for Xe/other atoms.

First, the best static (Stat) reference value for Xe chemical shift was computed at the center of the cavity at the SO-ZORA/BHandHLYP level. Thereafter the dynamical contribution (Dyn) due to Xe motion was estimated as a function of temperature *via* canonical *NVT* Monte Carlo statistical simulation averaging on three-dimensional surfaces of NR chemical shift and potential energy (see Fig. S21†). The effects due to periodic cage and its dynamics or different Xe loadings in neighboring cavities were not taken into account in these simulations.

The resulting overall chemical shift values are shown in Fig. 2C. Calculated chemical shifts and their dynamic contributions at different temperatures are shown in Tables S5 and S6.† At *T* = 300 K, the calculated ^129^Xe chemical shift in the cage cavity is only 22 ppm (total = Stat + Dyn = –21 ppm + 43 ppm), while it is 211 ppm (181 ppm + 30 ppm) in the smaller window cavity. We note that similar ^129^Xe chemical shift calculations for other cavity systems have proven to be in very good agreement with experiments,^20,26a^ implying good accuracy of current values as well. The relativity is an important phenomenon for the Xe chemical shift in CC3-R, since at SO-ZORA level it increases the cage and window shifts by *ca.* +3 ppm and +36 ppm, respectively. As the relativity has a larger role in the window cavity, it increases the chemical shift difference between the cavities by *ca.* +32 ppm (19%). While most of this difference is due to SR, also SO effects are important as their absolute contribution in the window cavity is notable, *ca.* 10 ppm, and about 29% of the total relativistic effect. This differs from the previously studied cavities, *e.g.*, Buckminster fullerene,^26*a*^ Fe_4_L_6_ metallosupramolecular,^20^ and fluorophenol clathrate cavities,^26*b*^ where the SO contributions were small, like in the CC3-R cage cavity. However, the shape of the 3D Xe shift surface and, hence, the thermal effect, are expected not to be affected much by relativity. Thus the temperature dependence of the Xe shift is well estimated around room temperature to be +0.064 ppm K^–1^ and +0.051 ppm K^–1^ for the cage and window cavities, respectively. The values are three to four times smaller than the slope of the experimental chemical shift observed for the ML and LL samples, while the slope for the HL sample has a different sign. Therefore, changes in the relative population of the cavities have a dominant role in explaining the temperature dependence of the shift.

The effect of loading, *i.e.*, the occupancy of nearby cavities, on the chemical shift was also modeled. The effect is quite similar in both cavities, *ca.* –14 ppm (see Table S7†). Therefore, it has an insignificant effect on the chemical shift difference between cavities. The structure of the CC3 material may also change with loading due to the flexibility of the material. This may have some influence on the chemical shift as well.^17*e*^

### Population of the cage and window cavities

Because CC3-R contains two binding sites for Xe, the cage and window cavities, it is justified to approximate the system by a two-site exchange model (see Fig. 4A).^32^ In the fast exchange region, the experimentally observed ^129^Xe chemical shift is the weighted average of the shifts in the cage and window cavities (*δ*_C_ and *δ*_W_):

1 *δ* = *X*_C_*δ*_C_ + *X*_W_*δ*_W_.

Eqn (1) makes it possible to extract the relative populations of xenon atoms in the cage and window cavities, *X*_C_ and *X*_W_, from the experimental chemical shifts, using the calculated *δ*_C_ and *δ*_W_ values (Fig. 2C). The resulting populations are shown in Fig. 2D. Relative population of Xe in the window cavity increases with loading: at room temperature, the populations are 39%, 47% and 68% for the LL, ML and HL samples, respectively. Consequently, the larger cage cavity is a more favorable adsorption site for xenon, and the window cavity becomes more occupied only at higher loadings.

### Exchange between the cage and window cavities

^129^Xe relaxation measurements provide deeper insight into exchange phenomena. At room temperature, *T*_1_ relaxation time of xenon adsorbed in the HL sample (48 s) is significantly longer than in the LL and ML samples (14 and 16 s, respectively), implying significantly reduced mobility of xenon (slower exchange between cage and window cavities) close to the sample saturation, because xenon atoms cannot pass each other in the small cavities. Variable temperature *T*_1_ data (Fig. S4†) show an interesting non-linear behavior of *T*_1_ of xenon in the HL sample with a maximum around 270 K, deviating from the linearly increasing *T*_1_ observed for the LL and ML samples.

**Fig. 3:**
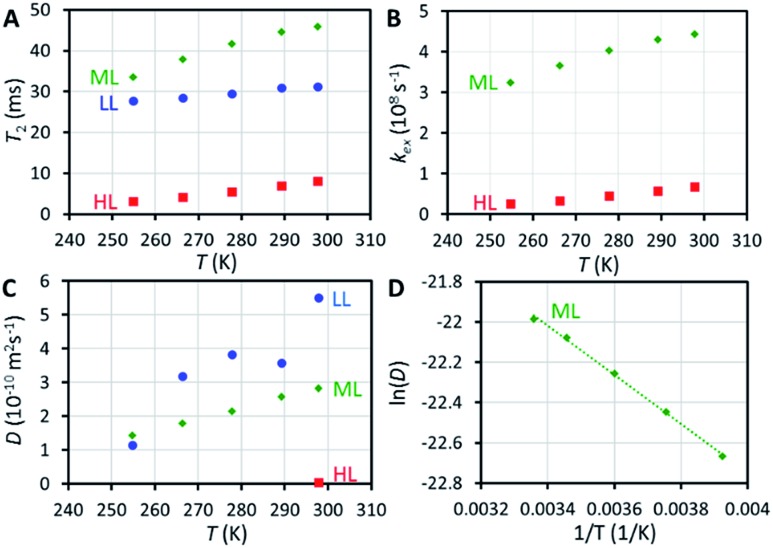
(A) ^129^Xe *T*_2_ relaxation times of xenon in CC3-R as a function of temperature. (B) Rates of xenon exchange between the window and cage cavities extracted from *T*_2_ data. (C) Diffusion coefficient of xenon in CC3-R as a function of temperature. The data of the LL sample is scattered because of the low signal-to-noise ratio in the experiment due to the low xenon concentration in the sample. (D) Arrhenius plot of the ML sample.

**Fig. 4:**
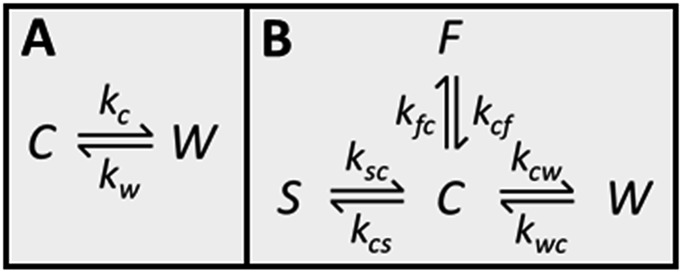
(A) Two-site exchange model used to extract populations from chemical shifts and exchange rates from *T*_2_ relaxation times. C refers to the cage cavity and W to the window cavity. Kinetic constants representing the exchange of xenon from cage to window cavity and *vice versa* are *k*_C_ and *k*_W_, respectively. (B) Four-site exchange model used in the analysis of the CEST spectra. S refers to the stuck window cavity and F to free gas.

*T*_2_ relaxation time of xenon in the HL sample (8 ms at room temperature) is significantly shorter than in the LL and ML samples (31 and 46 ms, respectively). Surprisingly, *T*_2_ value of the LL sample is between the values of the HL and ML samples. The slope of the *T*_2_ with respect to temperature is also smaller in the LL sample than in the ML and HL samples (see Fig. 3A). We interpret that the effect of interparticle exchange on *T*_2_ is significant in the LL sample due to the high mobility of xenon (revealed by the diffusion experiments described below), explaining the differing *T*_2_ behavior. Because the effect appears to be insignificant in the ML and HL samples, a two-site exchange model (Fig. 4A) can be used in the *T*_2_ relaxation time analysis. In the fast exchange limit the transverse relaxation rate constant is given by^33^

2 *R*_2_ = *X*_C_*X*_W_Δ*ω*^2^/*k*_ex_.

here Δ*ω* = *ω*_C_ – *ω*_W_ is the angular frequency difference between the cage and window cavity sites, and *k*_ex_ = *k*_C_ + *k*_W_, where *k*_C_ and *k*_W_ are the kinetic constants for the exchange from cage to window and *vice versa*, respectively (see Fig. 4A). Because *X*_C_, *X*_W_ and Δ*ω* are known based on the experimental and computational analysis of chemical shifts described above, the exchange rates *k*_ex_ can be estimated by inserting measured *T*_2_ values in eqn (2). The results are shown in Fig. 3B and Table S2 in ESI.† At room temperature, the exchange rate of xenon in the HL sample is *k*_ex_ = 6.8 × 10^7^ s^–1^, which is about six times smaller than that in the ML sample, *k*_ex_ = 4.4 × 10^8^ s^–1^. The rate increases with temperature for both samples.

### Four-site exchange

^129^Xe CEST spectra^18^ of the samples reveal additional details of the exchange processes and sample structure. In these experiments, the sample was first irradiated by a long continuous wave (CW) pulse (pulse length 5–10 s), followed by a hard π/2 excitation pulse and signal detection. The amplitudes of the signal as a function of the offset of the CW pulse, *i.e.*, the CEST spectra for LL, ML and HL samples are shown in Fig. 2B. In addition to the main dips at the same chemical shifts with the signals observed in the conventional spectra (Fig. 2A), all the CEST spectra include another dip around 211 ppm. The chemical shift of the dip matches perfectly with the shift of xenon in the window cavity predicted by the calculations (211.1 ppm). Therefore, we interpret that the dip arises from the window cavity. On the other hand, the perfect agreement may be considered to indicate also the high accuracy of the calculations. However, the dip has to arise from such window sites, from which xenon exchange to the cage cavities is slow, because separate signals can be observed in the CEST spectra only in the slow or intermediate exchange region, similarly to the conventional spectra.^18^ Therefore we call these cavities “stuck window cavities”. The population of the stuck window cavities must be very small, because their signal is not visible even in the conventional spectrum measured with a high number of scans, with signal-to-noise ratio (SNR) of 4000 (see Fig. S1†). The stuck window signal becomes observable in the CEST spectrum because of significant CEST signal amplification effect of a minor site, being several orders of magnitude.^18^

In the case of the HL sample, there is a third dip around 0.6 ppm, which is interpreted to arise from free xenon gas between the particles. In fact, a small free gas signal is observed at the same chemical shift also in the high SNR conventional spectrum (see Fig. S1†). The free gas signal is observable from the HL sample, because of two reasons: firstly, the amount of free gas is higher than in the other samples, and, secondly, the exchange between free and adsorbed sites is slower, because almost all the adsorption sites are occupied in the HL sample.

The CEST observations suggest that, instead of the two-site model, a four-site exchange model is more precisely characterizing the xenon exchange phenomena in CC3-R. As illustrated in Fig. 4B, the cage cavities are connected to three other sites: the window cavities, the stuck window cavities and the free gas pool. On the other hand, these three sites are not directly connected to each other, because xenon can move from one window cavity to another only *via* a cage cavity. When a free gas atom enters a CC3-R crystal, it has to arrive first in a cage cavity, because window cavities exist only in between the cage molecules. Simulated CEST spectra (see Fig. 2B and ESI†) suggest that the population of the stuck window cavities is between 0.02% and 0.3%, and the exchange rate between the stuck window and cage cavities is about 14 000 s^–1^. The stuck window cavities may arise from crystal defects, such as dislocations and grain boundaries, in CC3-R,^34^ and the population of these cavities may reflect the amount of defects in the material. Good agreement between the measured and simulated CEST spectra confirm that the exchange rates determined from the *T*_2_ values are reliable. The population of the free gas site is 0.9%, and the exchange rate between the free gas and cage cavities is about 14 000 s^–1^.

### Diffusion of xenon in CC3-R

NMR is one of the rare methods for measuring self-diffusion of molecules without an invasive tracer.^35^ We investigated the diffusion of xenon in CC3-R by ^129^Xe pulsed-field-gradient stimulated-echo (PGSTE)^36^ experiments, using bipolar gradients minimizing the effect of background gradients.^37^ Diffusion coefficient (*D*) of xenon in CC3-R is many orders of magnitude smaller than that of free xenon (5.3 × 10^–6^ m^2^ s^–1^),^38^ and it decreases significantly with loading: at room temperature, *D* is 5.5 × 10^–10^, 2.8 × 10^–10^ and 4.1 × 10^–12^ m^2^ s^–1^ for the LL, ML and HL samples, respectively (see Fig. 3C). *D* in the HL sample, which is almost saturated, is two orders of magnitude smaller than in the LL sample. This is a consequence of single-file nature of the diffusion:^39^ xenon atoms cannot pass each other in the small cavities, and therefore high loading significantly restricts the moving of xenon atoms. Mean distance diffused by xenon atoms in the diffusion time *Δ*, characterized by the square root of the mean-square displacement (MSD), (2*DΔ*)^1/2^ = 2–3 μm, is on the same order with the size of smallest particles in the sample (particle sizes between 0.5 and 50 μm). Because of this, the second diffusion component was observed (see Fig. S12 and S13†), arising from interparticle exchange, and characterized by the diffusion coefficient of about 2 × 10^–9^ m^2^ s^–1^. On the other hand, because most of the particles are much larger than the mean diffusion distance, the first diffusion coefficient reflects pure intraparticle diffusion.

The activation energy for diffusion of xenon in the ML sample was determined by fitting an Arrhenius function to the variable temperature *D* values (see Fig. 3D and S14†). The resulting activation energy is (10.1 ± 0.3) kJ mol^–1^. Chen *et al.* ^9^ fitted stretched exponential model to adsorption isotherms, and obtained activation energies ranging from 6.0 to 17.5 kJ mol^–1^ for xenon loading of 0 to 2.0 mmol g^–1^. Bearing in mind the loading of the ML sample (0.48 mmol g^–1^), our NMR result is in good agreement with the adsorption measurements.

In long, straight, cylindrical channels, which are not interconnected, single-file diffusion MSD is proportional to square root of time 
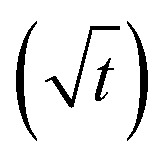
 instead of normal Fickian *t* dependence,^39^ leading to an apparent *D*, which is inversely proportional to the square-root of the diffusion time, *Δ* ^–1/2^. The value of *D* of the HL sample, however, turned out to be independent of *Δ* within experimental error. Because CC3-R is composed of three-dimensional interconnected network of pores, instead of straight cylinders, the single-file diffusion is not present itself in the same manner as in the case of non-interconnected straight channels.

### Equilibrium between bound and free xenon

The equilibrium of xenon between bound and free gas phases was studied in the ML and HL samples at the temperature range of 255–298 K by comparing the integrals of ^129^Xe signal measured from the CC3-R and gas regions of the sample (see ESI†). The gas region was measured by turning the sample upside down (a piece of glass wool prevented the moving of the cage material). The mole fractions of xenon in the gas phase and cage were calculated from the integrals, using the known overall amounts of Xe and CC3-R added into the samples. Van't Hoff analysis of the equilibrium constants yielded the following values for the changes of Gibbs free energy, enthalpy and entropy, for ML and HL samples, respectively: Δ*G* = –(30 ± 4) and –(25.9 ± 1.4) kJ mol^–1^, Δ*H* = –(9 ± 2) and –(1.8 ± 0.7) kJ mol^–1^ and Δ*S* = (71 ± 6) and (81 ± 3) J mol^–1^ K^–1^. The binding is both enthalpy and entropy driven. Enthalpy is significantly lower for the HL sample.

### Equilibrium between xenon in the cage and window cavities

Based on the populations of the cage and window cavities shown in Fig. 2D, we were able to investigate the equilibrium of xenon between the cage and window cavities (see ESI†). In the cases of the LL and ML samples, the cage cavity binding is favored, with the changes of Gibbs free energy, enthalpy and entropy of Δ*G* = –(4.4 ± 0.3) and –(2.5 ± 0.4) kJ mol^–1^, Δ*H* = –(3.7 ± 0.2) and –(1.6 ± 0.2) kJ mol^–1^ and Δ*S* = (2.5 ± 0.6) and (2.9 ± 0.6) J mol^–1^ K^–1^, respectively. However, in the case of the almost saturated HL sample, the binding affinities of these sites are almost equal, and the corresponding values are Δ*G* = +(0.9 ± 0.6) kJ mol^–1^, Δ*H* = +(8.0 ± 0.3) kJ mol^–1^ and Δ*S* = (24 ± 1) J mol^–1^ K^–1^.

## Conclusions

Combined state-of-the-art experimental and computational ^129^Xe analysis provided extraordinarily versatile inside information on the adsorption of xenon in a crystalline porous organic cage, CC3. It enabled us to determine the populations of the cage and window cavities as well as exchange rates between them, diffusion coefficients and activation energy of diffusion, and thermodynamic parameters of the equilibrium between the bound and free xenon as well as between xenon in the cage and window cavities. Furthermore, the analysis revealed a minor “stuck” window cavity site, which may be associated with crystal defects. The analysis improves significantly the understanding of the extraordinarily high adsorption of noble gases in the organic cages, facilitating their use in gas separation as well as other conceivable applications, such as biosensor applications.
