# Supplementary material for: Inside information on xenon adsorption in porous organic cages by NMR
Source: Chem Sci. 2017 Jun 14;8(8):5721–7. doi: 10.1039/c7sc01990d (PMC5621166; doi:10.1039/c7sc01990d)
Supplement: Supplementary file 1 [file SC-008-C7SC01990D-s001.pdf]

Electronic Supplementary Material (ESI) for Chemical Science.
This journal is © The Royal Society of Chemistry 2017

Electronic Supporting Information (ESI) to the article

Inside Information on Xenon Adsorption in Porous Organic Cages by NMR

*Sanna Komulainen, Juho Roukala, Vladimir V. Zhivonitko, Muhammad Asadullah Javed, Linjiang Chen, Daniel Holden, Tom Hasell, Andrew Cooper, Perttu Lantto and Ville-Veikko Telkki**

Contents

1. Experimental 2
1.1 Sample preparation 2
1.2 NMR experiments 2
2. Experimental results 4
2.1 ^{129}Xe spectra 4
2.2 Relaxation measurements 5
2.3 Exchange rates 6
2.4 CEST spectra 8
2.5 Four-site exchange model for the simulations of the CEST spectra 9
2.6 Diffusion measurements 11
2.7 Equilibrium between bound and free xenon 13
2.8 Equilibrium between xenon in the cage and window cavities 15
3. Computational modeling 15
3.1 Cavity model structures 16
3.2 DFT calculations 16
3.3 Computed chemical shifts at the centers of the cavities 17
3.4 DFT functional tests 18
3.5 Relativistic contributions to Xe chemical shift 19
3.6 Exchange-correlation kernel in ADF 2016 20
3.7 Dynamical modeling and total estimate of the ^{129}Xe chemical shift 20
3.8 Loading effect on Xe shifts in cage and window cavities 23
4. References 23

1. Experimental

1.1 Sample preparation

To synthesize **CC3**-*R*, dichloromethane (DCM, 100 mL) was added slowly onto solid 1,3,5-triformylbenzene (5.0 g, 30.86 mmol) without stirring at room temperature. Trifluoroacetic acid (100 µL) was added directly to this solution as a catalyst for imine bond formation. Finally, a solution of (R,R)-1,2-diaminocyclohexane (5.0 g, 44.64 mmol) in DCM (100 mL) was added. The unmixed reaction was covered and left to stand. Over 5 days, all of the solid triformylbenzene was consumed, and octahedral crystals grew on the sides of the vessel. The crystalline product was removed by filtration and washed with 95% ethanol/5% DCM. Yield: 6.5 g, 83%. ^{1}H NMR ($CDCl_3$): δ 8.15 (s, CHdN, 12H), 7.89 (s, ArH, 12H), 3.33 (m, CHN, 12H), 1.9_1.4 (m, CH2, 48H) ppm. ^{13}C NMR ($CDCl_3$): δ 159.1, 136.7, 129.5, 74.7, 33.0, 24.4 ppm. MS (ES+): 1118 ([M+H]+). Accurate mass calculated for C72H85N12: 1117.7020. Found: 1117.7065.

The Xe@CC3-R samples were prepared in the following way: The CC3-R cage material was transferred into a 5-mm medium wall NMR tube. The NMR tube was then connected to a vacuum line and dried overnight at 106 °C under vacuum. A proper volume of Xe gas (^{129}Xe isotope enriched 91%) was then transferred into the NMR tube (to get required amount of ^{129}Xe for the HL sample, at the end the rest of Xe was condensed in the sample by liquid N_2). Finally, the NMR tube was immersed in liquid N_2, and the tube was sealed with a flame. The molar ratio between Xe and CC3-R material (Xe:CC3-R) in the NMR tube was calculated to be 0.1:1 for the LL, 0.52:1 for the ML and 3.3:1 for the HL samples. In the ML sample 99 % of Xe and in the HL sample 80 % of Xe are bound by the CC3-R material at RT giving 0.025 bar and 3.9 bar xenon gas pressures inside the tube, respectively. Based on this, the actual molar ratio of bound ^{129}Xe in the HL sample is 2.4:1.

1.2 NMR experiments

^{129}Xe NMR experiments were carried out using Bruker Avance III 600 spectrometer with the magnetic field of 14.1 T and ^{129}Xe frequency of 166 MHz. A 5-mm BBFO probe with z-gradients was used in the experiments. Temperature series were measured with a temperature stabilization time of 60 min. The reading temperatures were calibrated with standard Bruker samples. ^{129}Xe chemical shifts were referenced with respect to low pressure Xe gas. Some broad temperature range experiments, which required N_2 cooling, were performed using Bruker Avance III 300 spectrometer with the magnetic field of 7.1 T and ^{129}Xe frequency of 83.0 MHz.

NMR spectra. Basic ^{129}Xe spectra were measured using pulse angle 90º with 1 scan. For the HL sample, a high SNR spectrum with 24576 scan using 15º pulse angle was recorded as well at room temperature. The equilibrium of bound and free xenon gas was studied by measuring ^{129}Xe spectra both from the CC3-R and gas regions of the sample. The gas region was measured by turning the

sample upside down (a piece of glass wool prevented the moving of the cage material). The ML spectra were accumulated with 64 scan with 400 recycling delay and the HL spectra only with 1 scan.

T_1 relaxation experiments. T_1 relaxation times of ^{129}Xe were measured using inversion recovery pulse sequence. The recycling delays were 80, 80 and 150 s, and the number of accumulated scans was 1, 2 and 1 for the LL, ML and HL samples, respectively.

T_2 relaxation experiments. T_2 relaxation times of ^{129}Xe were measured using CPMG pulse sequence. The recycling delays were 60, 60 and 200 s, and the number of accumulated scans was 8, 4 and 2 for the LL, ML and HL samples, respectively.

CEST experiments. In the ^{129}Xe CEST NMR experiments, the B_1 field strength of the CW varied between 5.7 and 30 μT (power from 1 to 30 mW). The CW pulse length varied from 5 to 15 s. The recycling delays and number of accumulated scans were 42, 70 and 150 s, and 16, 1 and 1 for the LL, ML and HL samples, respectively.

Diffusion experiments. ^{129}Xe diffusion measurements were carried out using a PGSTE experiment with bipolar gradients. The recycling delays and number of accumulated scans were 60, 55 and 150 s, and 16, 1 and 8 for the LL, ML and HL samples, respectively. Diffusion delay Δ was varied from 0.05 to 25 s and length of the gradient pulse δ from 0.3 to 2 ms.

2. Experimental results

2.1 ^{129}Xe spectra

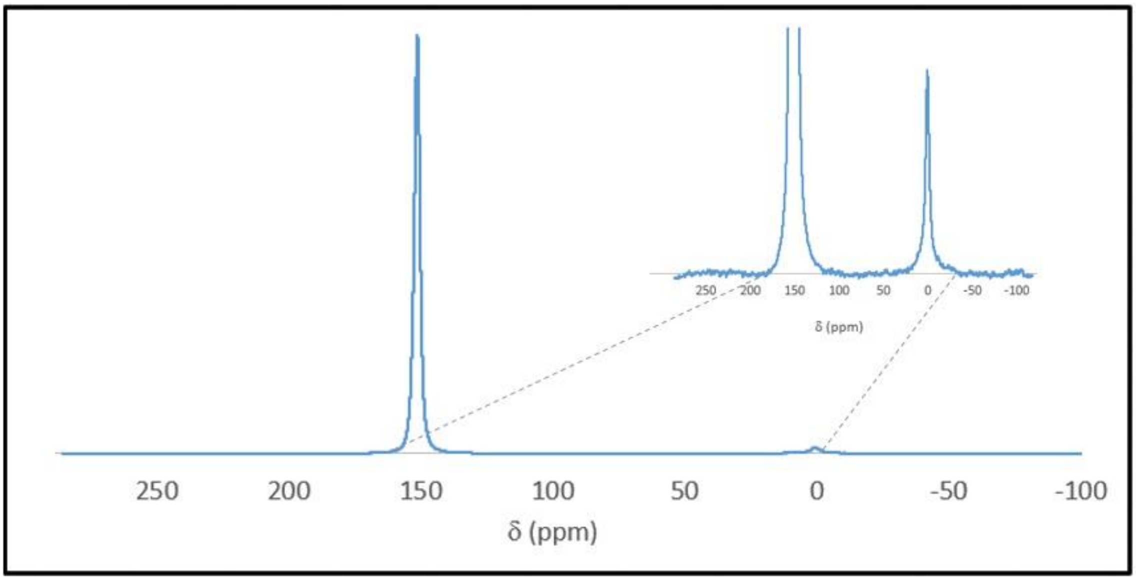

Figure S1. High SNR spectrum of the HL sample measured with 24576 scans using a pulse angle of 15° at 14.1 T. A small free gas signal is visible around 0.6 ppm. No window or cage cavity signal is visible around 211 or 22 ppm, although SNR is about 4000.

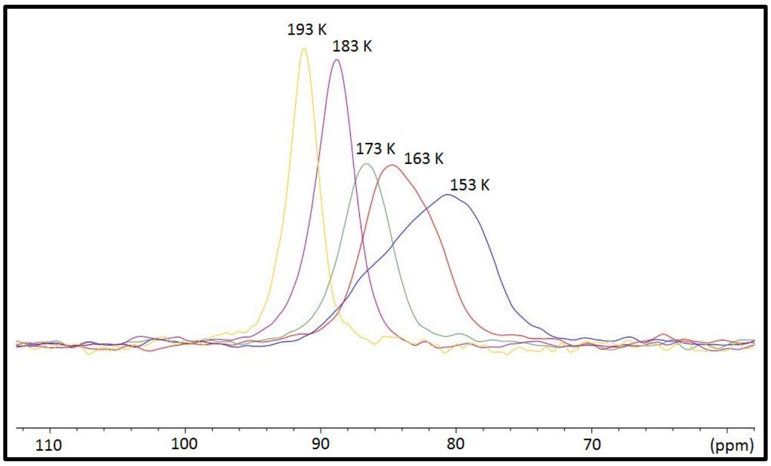

Figure S2. Spectra of ML sample at temperature range of 153-193 K measured at 7.1 T. At the lowest temperatures, the signal becomes broader because of gradual transition from fast to intermediate exchange rate region. Because separated signals from the cage and window cavities around 20 and 200 ppm are not resolved, the system is not in the slow exchange region even at the lowest temperature.

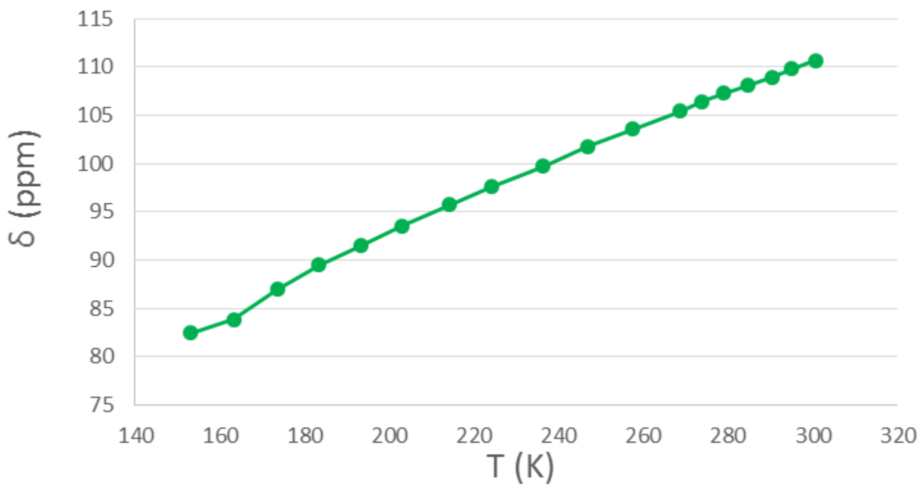

Figure S3. Chemical shift of the ML sample in a broad temperature range, measured at 7.1 T. The shift increases quite linearly with temperature.

2.2 Relaxation measurements

Table S1. T_1 and T_2 relaxation times of ^{129}Xe in CC3-R samples at variable temperature, measured at 14.1 T.

T(K)	T_1 (s)			T_2 (ms)		
	LL	ML	HL	LL	ML	HL
298	13.7	16.6	48.2	31.1	45.9	8.1
289	12.7	15.8	53.8	30.9	44.5	6.9
278	11.6	14.4	58.9	29.4	41.7	5.5
266	11.3	13.4	59.8	28.4	37.8	4.2
255	8.9	12.6	56.4	27.7	33.6	3.2

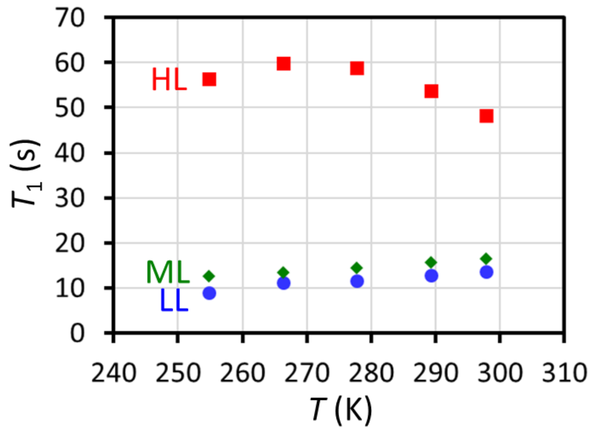

Figure S4. T_1 relaxation times of ^{129}Xe in CC3-R samples at variable temperature, measured at 14.1 T.

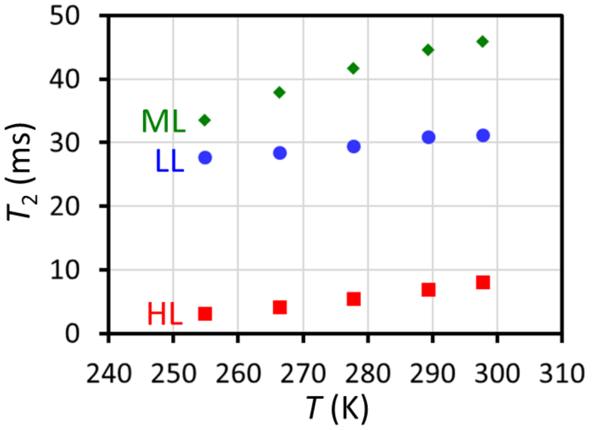

Figure S5. T_2 relaxation times of ^{129}Xe in CC3-R samples at variable temperature, measured at 14.1 T.

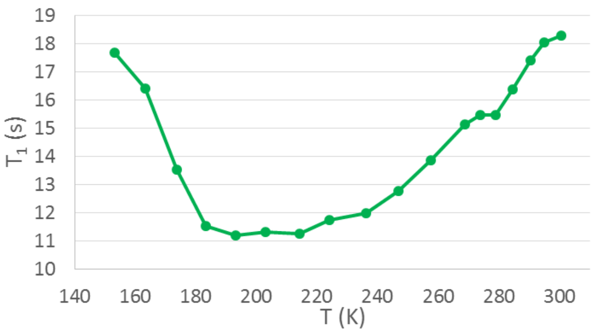

Figure S6. T_1 relaxation time of ^{129}Xe in the ML sample as a function of temperature measured at 7.1 T. Between 250 and 300 K, T_1 values are close to the values measured at 14.1 T (see Figure S4 and Table S1). There is a minimum in the curve around 200 K. The discontinuity in the T_1 trend around 280 K is a consequence of a hysteresis effect because of the restart of the experiment.

2.3 Exchange rates

$$C \underset{k_w}{\overset{k_c}{\rightleftharpoons}} W$$

Figure S7. Simple two-site exchange model used in the analysis. C refers to the cage cavity and W to the window cavity. Kinetic constants representing the exchange of xenon from cage to window cavity is k_c, and k_w is the constant for opposite exchange.

Table S2. Exchange rates k_c, k_w and k_{ex} ($k_{ex} = k_c + k_w$) of xenon between the cage and window cavities determined by substituting experimentally determined T_2 relaxation times (see Table S1), calculated chemical shifts of Xe in the cage and window cavities (see Figure 2 C) and populations X_c and X_w (shown in the two columns on the right and Figure 2 D) into Eq. 2. The populations were calculated from the chemical shifts using Eq. 1.

T(K)	k_c (10^8 s^{-1})			k_w (10^8 s^{-1})			k_{ex} (10^8 s^{-1})			X_c			X_w		
	LL	ML	HL	LL	ML	HL	LL	ML	HL	LL	ML	HL	LL	ML	HL
298	1.13	2.09	0.46	1.75	2.35	0.21	2.88	4.43	0.68	0.61	0.53	0.32	0.39	0.47	0.68
289	1.18	1.99	0.39	1.75	2.31	0.18	2.85	4.30	0.57	0.61	0.54	0.31	0.39	0.46	0.69
278	1.02	1.86	0.31	1.67	2.18	0.14	2.70	4.03	0.45	0.62	0.54	0.31	0.38	0.46	0.69
266	0.95	1.66	0.24	1.63	2.00	0.10	2.58	3.66	0.34	0.63	0.55	0.30	0.37	0.45	0.70
255	0.89	1.45	0.18	1.60	1.79	0.08	2.49	3.25	0.26	0.64	0.55	0.29	0.36	0.45	0.71

2.4 CEST spectra

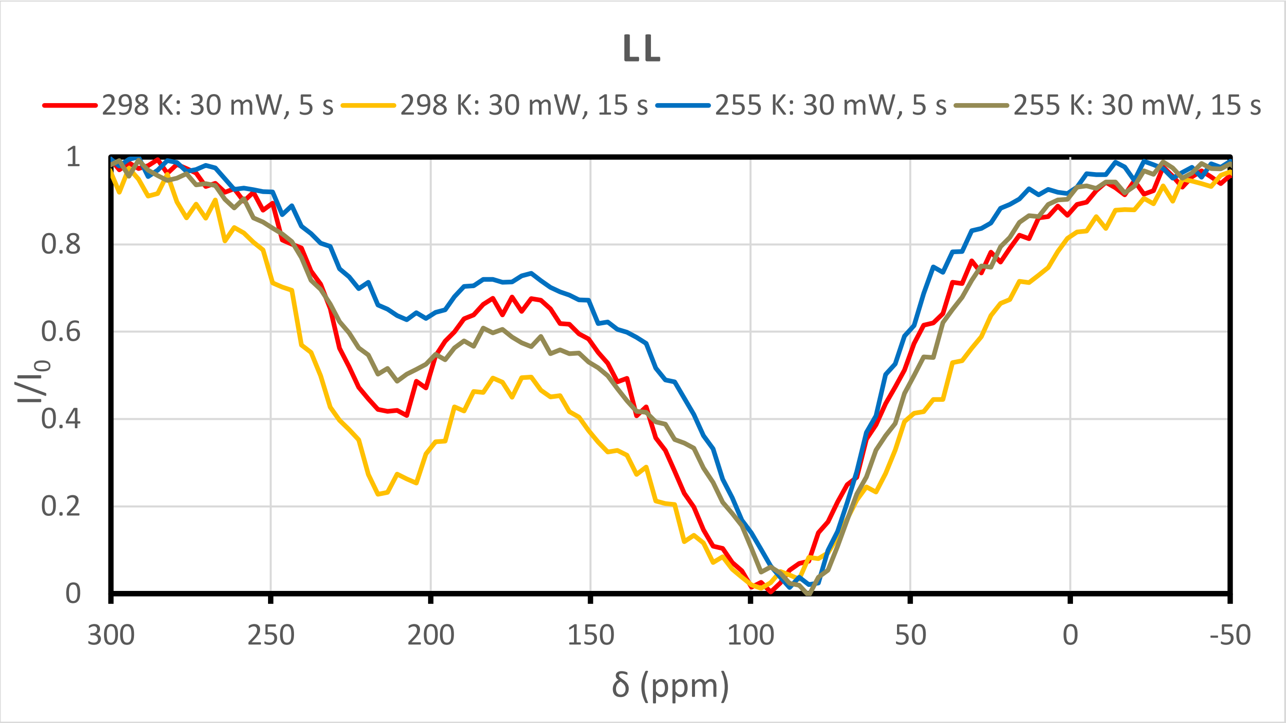

Figure S8. [129]Xe CEST spectra of the LL sample with variable temperature, CW pulse power and length shown in the figure, measured at 14.1 T.

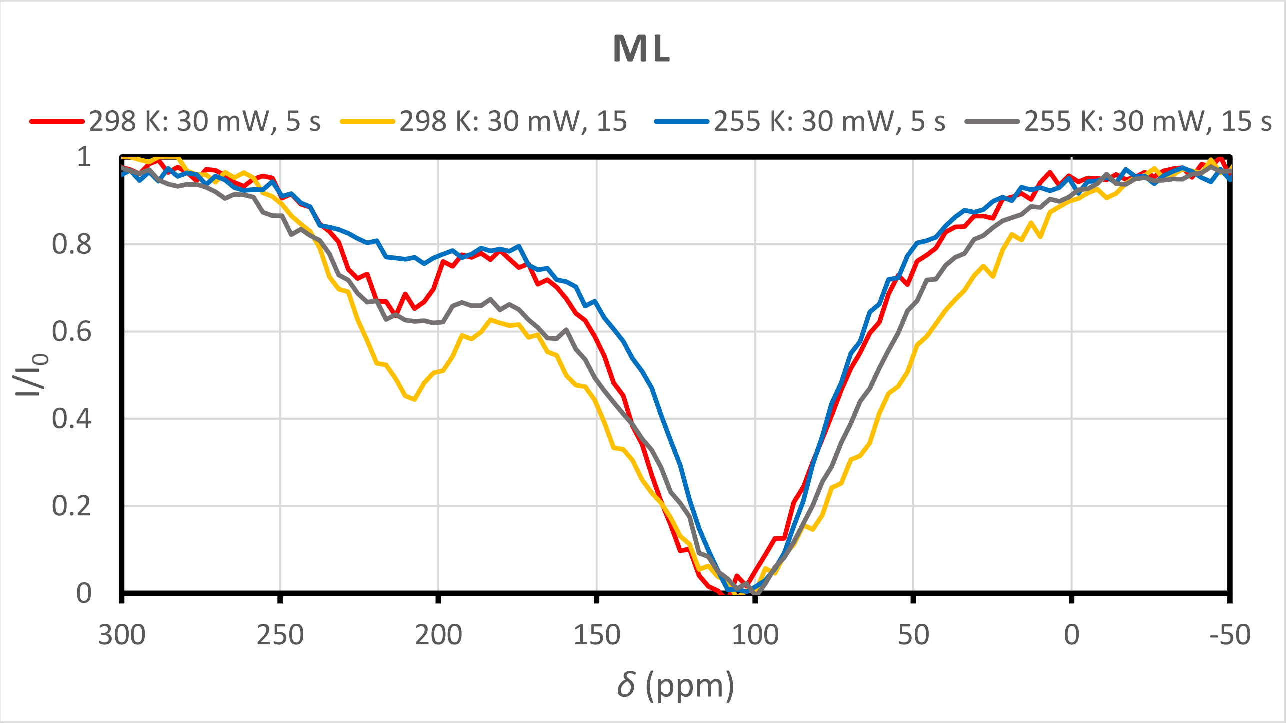

Figure S9. [129]Xe CEST spectra of the ML sample with variable temperature, CW pulse power and length shown in the figure, measured at 14.1 T.

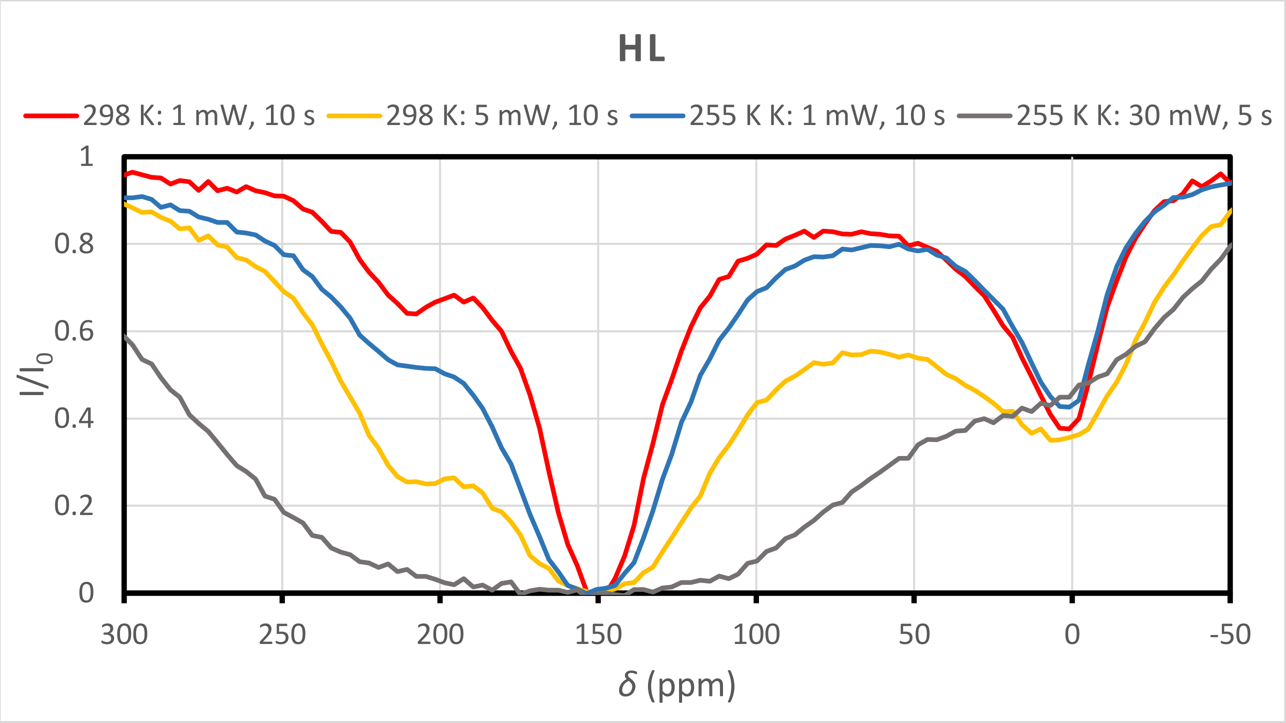

Figure S10. ^{129}Xe CEST spectra of the HL sample with variable temperature, CW pulse power and length shown in the figure, measured at 14.1 T.

2.5 Four-site exchange model for the simulations of the CEST spectra

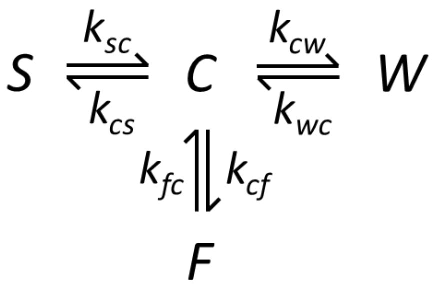

Figure S11. Four-site exchange model used in the analysis of the CEST spectra. C refers to the cage cavity, W to the window cavity, S to the stuck window cavity and F to free gas.

Simulations of CEST spectra for the four-site exchange were performed by using four sets of Bloch's equations [1] coupled by kinetic terms according to Figure S11 (k_{sc},k_{cs},k_{fc},k_{cf},k_{cw},k_{wc}). The resulting set of the equations was solved numerically in MATLAB for different resonance offsets leading to CEST spectra (the normalized ^{129}Xe NMR signal amplitude as a function of the resonance offset). Required parameters in the simulation were either known from experiment, or optimized, or estimated from quantum chemistry methods. Nutation frequencies were determined from experimental $\pi/2$-pulses at a given power level. T_1 and T_2 times were measured experimentally, and it was assumed that there is no difference in these quantities between cage and window sites, Table

S1. Chemical shifts were estimated from quantum chemistry methods as describe in the main text. k_{cw} and k_{wc} were determined from T_2 measurements (see main text), whereas k_{sc}, k_{cs}, k_{fc} and k_{cf} were optimized iteratively to get best match between experimental and simulated CEST spectra. The optimized constants were used to calculate the populations of "stuck" and free Xe in the samples.

Table S3. Values of kinetic constants used in simulations of the CEST spectra shown in Figure 2 B, based on the four-site exchange model illustrated in Figure S11.

	LL	ML	HL
k_{cw} (s^{-1})	$1.11 \cdot 10^8$	$2.01 \cdot 10^8$	$4.64 \cdot 10^7$
k_{wc} (s^{-1})	$1.77 \cdot 10^8$	$2.42 \cdot 10^8$	$2.14 \cdot 10^7$
k_{cs} (s^{-1})	14	5.4	108
k_{sc} (s^{-1})	15000	13500	13500
k_{cf} (s^{-1})	0	0	400
k_{fc} (s^{-1})	0	0	14000

Table S4. Values of populations used in simulations of the CEST spectra shown in Figure 2 B, based on the four-site exchange model illustrated in Figure S11.

	LL	ML	HL
X_c	0.61	0.55	0.31
X_w	0.39	0.45	0.68
X_s	0.00057	0.00022	0.0025
X_f	0	0	0.0089

2.6 Diffusion measurements

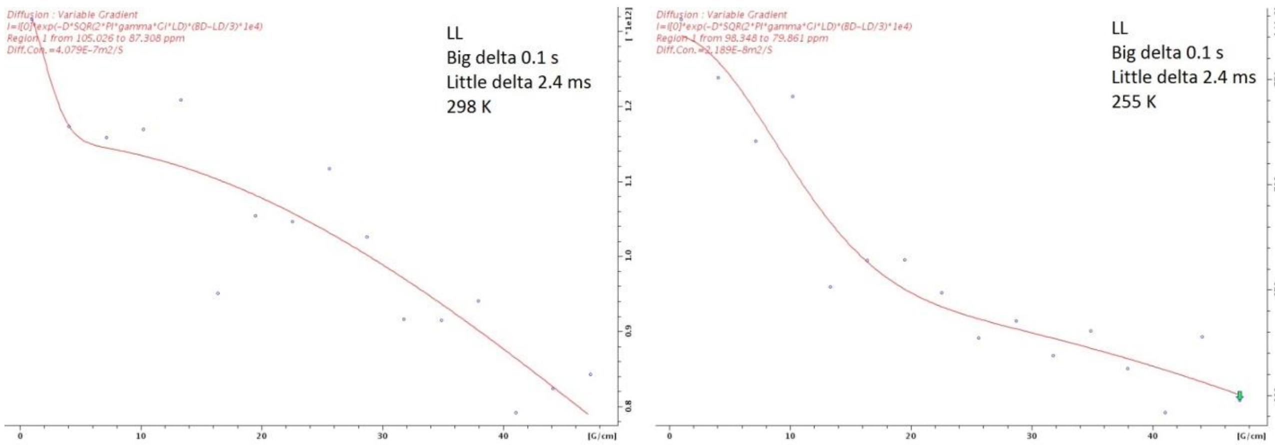

Figure S12. Integral of the signal in the diffusion experiments as a function of gradient strength spectra for the LL sample at 298 K (left) and 255 K (right). Two components are clearly visible. The faster decaying component arises from interparticle diffusion, the slower component from diffusion inside the CC3-R particles.

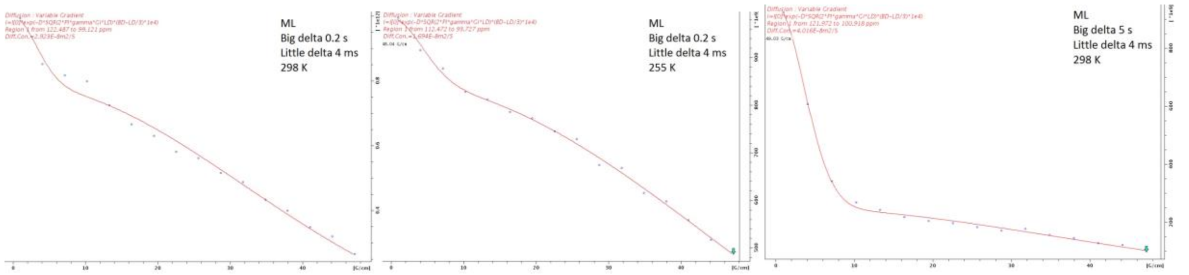

Figure S13. Integral of the signal in the diffusion experiments as a function of gradient strength spectra for the ML sample at 298 K (left), 255 K (middle) and 298 K (right). The diffusion delay is longer (5 s) in the right experiment than in others (0.2 s). Two components are clearly visible. The faster decaying component arises from interparticle diffusion, the slower component from diffusion inside the CC3-R particles. Interparticle diffusion is more dominant with the longer diffusion delay.

The temperature dependence of the diffusion coefficient is represented by the Arrhenius function

$$D = D_0 \exp\left(\frac{-E_D}{RT}\right), \tag{S1}$$

where D_0 is the pre-exponential factor, E_D is the activation energy for diffusion, and R is the gas constant. Taking a natural logarithm from each side, Eq. S1 becomes the following form:

$$\ln D = \ln D_0 - \left(\frac{-E_D}{R} \frac{1}{T} \right). \qquad \text{(S2)}$$

Plot of ln*D* with respect to 1/*T* is shown in Figure S14. The slope ($-E_D/R$) yields E_D = (10.1 ± 0.3) kJ/mol for the ML sample.

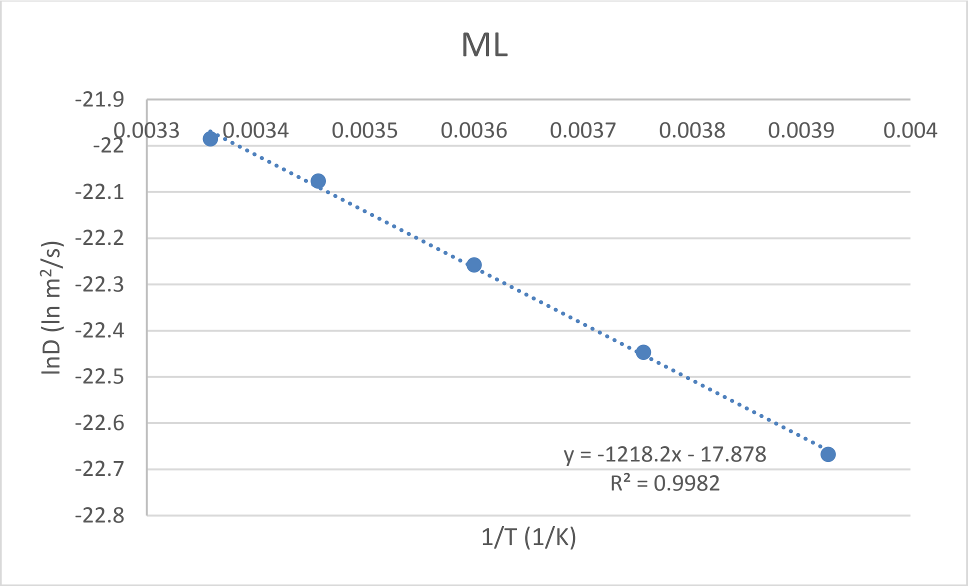

Figure S14. Arrhenius plot.

2.7 Equilibrium between bound and free xenon

Chemical equation describing equilibrium of bound and free xenon is

$$Xe_f + B_f = Xe_b, \qquad \text{(S3)}$$

where Xe_f is the amount of free Xe atoms, B_f is the amount of free binding sites in CC3-R and Xe_b is the amount of bound Xe atoms. Because there are three binding sites per each cage molecule in the material (one cage cavity site and two window cavity sites), $B_f \leq 3C_n$, where C_n is the amount of cage molecules in the sample. On the other hand, $B_f = 3C_n - Xe_b$. Therefore, the equilibrium constant is

$$K_N^{fb} = Xe_b/(Xe_fB_f) = Xe_b/[Xe_f(3C_n - Xe_b)]. \qquad \text{(S4)}$$

The relative amounts of Xe in the free and bound gas sites were determined by integrating the NMR signals measured from the CC3-R and free gas regions (see Figures S15 and S16, in the latter case the sample was upside down), and the resulting values were converted into absolute amounts (numbers) by using known overall amounts of Xe and CC3-R added into the samples. The latter quantities were obtained from the total volumes of free gas and the mass of CC3-R in the sample tubes. The values were substituted into Eq. S4 in order to determine the equilibrium constants. Thermodynamic parameters were extracted from the data by a standard van't Hoff plot analysis, resulting in the following values for the changes of Gibbs free energy, enthalpy and entropy, for ML and HL samples, respectively: $\Delta G = -(30\pm4)$ and $-(25.9\pm1.4)$ kJ/mol, $\Delta H = -(9\pm2)$ and $-(1.8\pm0.7)$ kJ/mol and $\Delta S = (71\pm6)$ and (81 ± 3) J/mol·K.

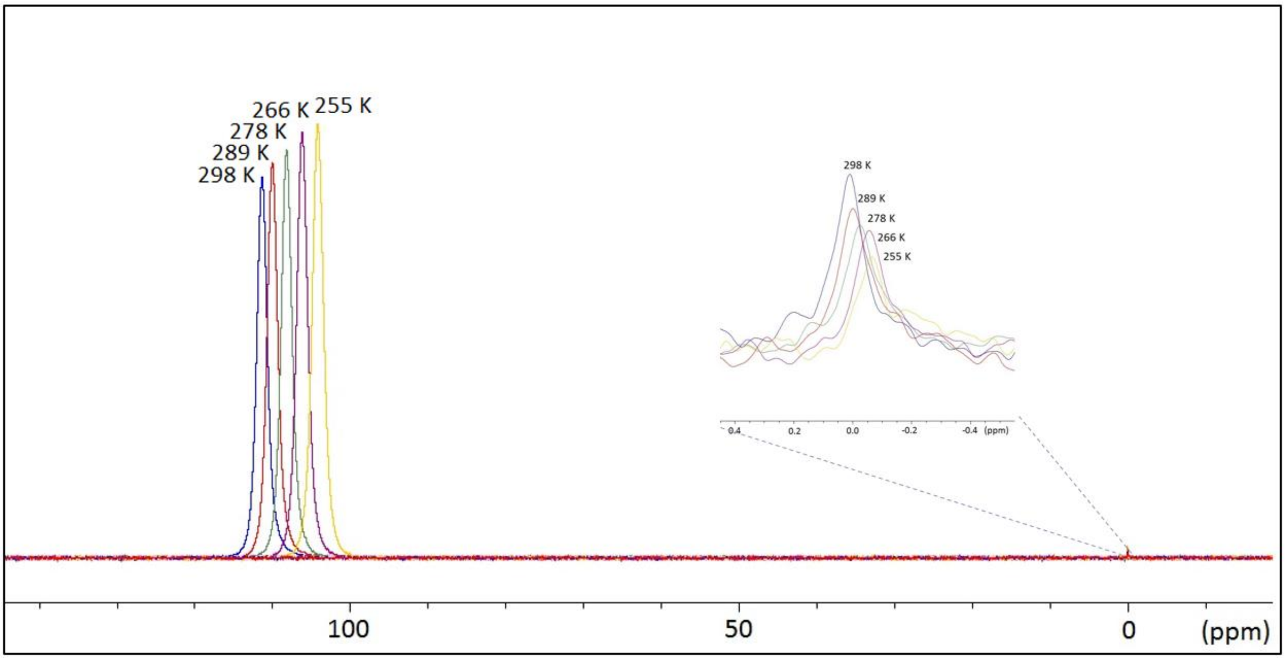

Figure S15. ^{129}Xe spectra measured from the CC3-R region (signals on the left) and free gas region (sample upside down, signals on the right) in the ML sample at variable temperature for the bound and free xenon equilibrium analysis.

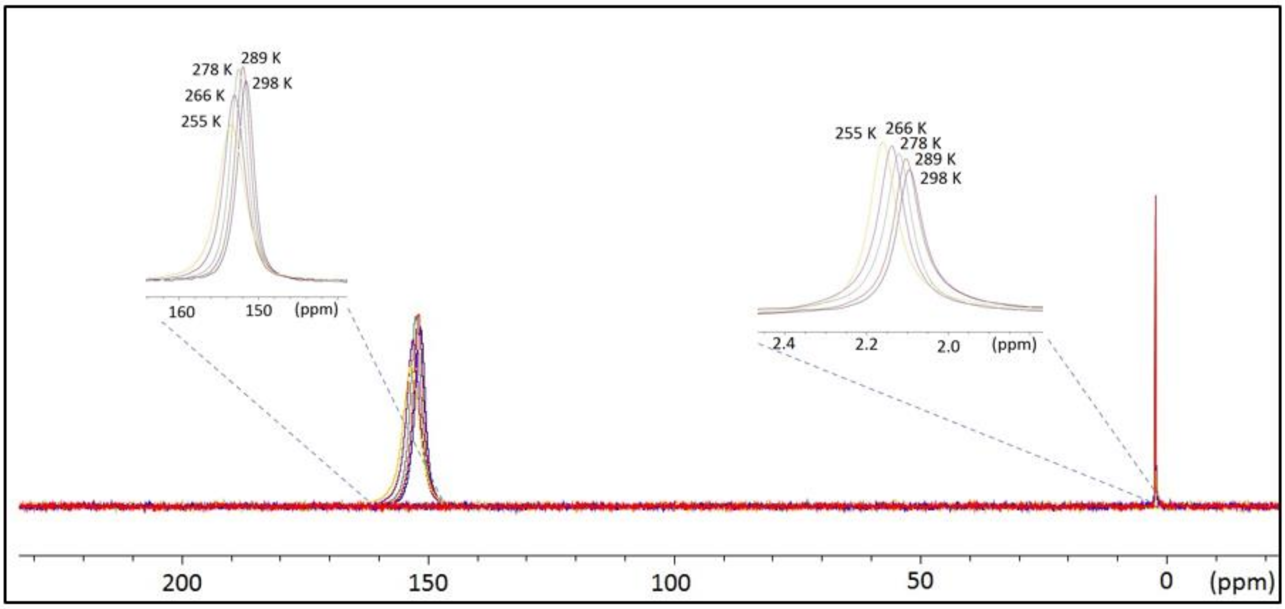

Figure S16. ^{129}Xe spectra measured from the CC3-R region (signals on the left) and free gas region (sample upside down, signals on the right) in the HL sample at variable temperature for the bound and free xenon equilibrium analysis.

2.8 Equilibrium between xenon in the cage and window cavities

Chemical equation describing equilibrium of xenon between the cage and window cavities is

$$C_f + Xe_w = W_f + Xe_c, \qquad (S5)$$

where C_f the amount of free cage binding sites, Xe_w is the amount of xenon atoms in window cavities, W_f is the amount of free window binding sites, and Xe_c is the amount of Xe atoms in the cage cavities. Because there are one cage cavity and two window cavities per each cage, $C_f \leq C_n$ and $W_f \leq 2C_n$, where C_n is the amount of cages in the sample. On the other hand, $C_f = C_n - Xe_c$ and $W_f = 2C_n - Xe_w$. Therefore, the equilibrium constant is

$$K_p^{cw} = W_f Xe_c/(C_f Xe_w) = (2C_n\text{-}Xe_w)Xe_c/[(C_n\text{-}Xe_c)Xe_w]. \qquad (S6)$$

Using the populations of the cage and window cavities shown in Figure 2 D and Table S2, the equilibrium constants were calculated by Eq. S6, and thermodynamic parameters were determined by a standard van't Hoff analysis. In the cases of the LL and ML samples, the cage cavity binding is favored, with the changes of Gibbs free energy, enthalpy and entropy of ΔG = -(4.4±0.3) and -(2.5±0.4) kJ/mol, ΔH = -(3.7±0.2) and -(1.6±0.2) kJ/mol and ΔS = (2.5±0.6) and (2.9±0.6) J/mol·K, respectively. However, in the case of the HL sample, the corresponding values are ΔG = +(0.9±0.6) kJ/mol, ΔH = +(8.0±0.3) kJ/mol and ΔS = (24±1) J/mol·K, implying that window cavity binding is favored close to sample saturation.

3. Computational modeling

As xenon is an ideal guest for supramolecular systems (chemically inert nature with easily polarized electron cloud), it also opens up interesting possibilities through the modeling of the atomic-scale dynamics of the guest, as was recently demonstrated in Ref. 2 for Xe in an iron-based cage. Xe NMR has also been utilized in studying the structure of ionic liquids [3] by combining classical molecular dynamics with relativistic density functional theory (DFT) calculations. The dynamic, solvent, and relativistic effects to the average Xe chemical shift have been studied in Xe@C60 dissolved in benzene [4], demonstrating that, while relatively small in comparison to the nonrelativistic static model, these effects are essential for obtaining good agreement with experiments. Whereas NMR experiments yield the true time-averaged point of reference, the theoretical model is able to provide minute details of, *e.g.*, the three-dimensional atomic positions as well as potential energy and chemical shift surfaces, which allow distinguishing the environment of the guest Xe as well as deducing how and why the NMR parameters are accumulating to the observed values in a given temperature.

3.1 Cavity model structures

The crystal structure of the solid material studied in this work consists of hollow cavities formed by hexagonal carbon rings, connected to each other by carbon and nitrogen atoms to form substructures of tetrahedral symmetry. Molecular density functional theory (DFT) modeling was based on three fixed-geometry cavity structures that represent the essential features of the two sites that xenon occupies. The smallest model (dubbed cage, see Figure S17) comprises of a single hollow $N_{12}C_{72}H_{84}$ structure with tetrahedral symmetry and four openings that, in the real crystal structure, act as gateways to other such hollow cavities. The slightly larger model (dubbed window, $N_{24}C_{144}H_{168}$) consists of two such adjacent tetrahedral units, with the interesting cavity being the tunnel between the two units. In both models, only a single ^{129}Xe was present, positioned at the center of the corresponding cavity for static calculations. The largest model is made of five tetrahedral cage units ($N_{60}C_{360}H_{420}$) and used to study the loading effects with different occupations of cage and window cavities.

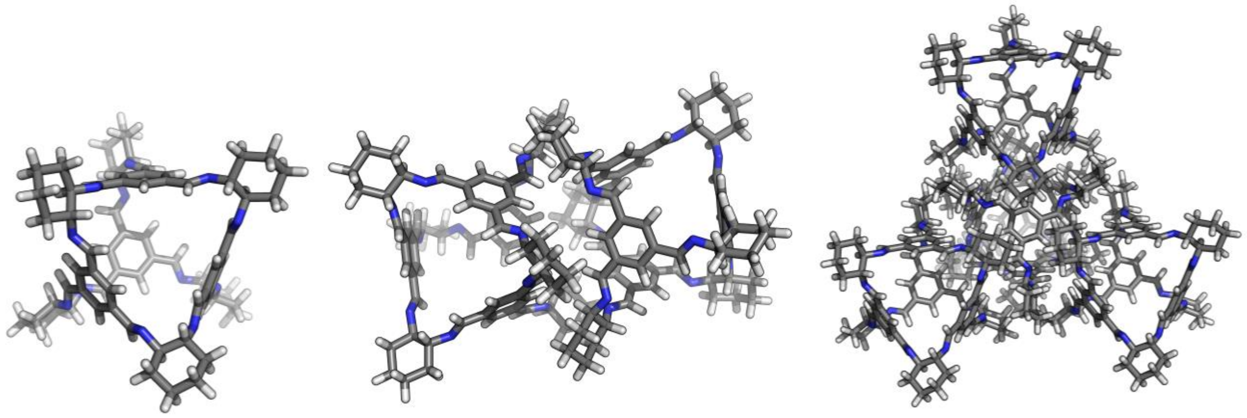

Figure S17. Models of the cage cavity ($N_{12}C_{72}H_{84}$, left), the window cavity made of two cage cavities ($N_{24}C_{144}H_{168}$, center), and the cluster of five cage cavities ($N_{60}C_{360}H_{420}$, right).

3.2 DFT calculations

Density functional theory calculations were performed with the hybrid BHandHLYP functional [5−7], using the Turbomole [8] code to obtain energy and chemical shift (CS) data for the dynamics simulations at the nonrelativistic (NR) level. Amsterdam Density Functional (ADF) [9−11] program package was used for relativistic calculations at the zeroth-order regular approximation (ZORA) [12−16] level of theory including either scalar relativistic only (SR-ZORA) or both scalar and spin-orbit relativistic (SO-ZORA) effects and using a Gaussian nuclear model [17]. ADF version 2014 was used for all calculations except for testing the newer exchange-correlation kernel available in version 2016 (*vide infra*). The DFT-D3 dispersion correction [18] was used in potential energy calculations. All-electron co-r[2,19,20]/def2-SVP[21] (Turbomole) and jcpl/TZP (ADF) basis sets were used for Xe/other atoms. The dynamical contributions to the ^{129}Xe CS were obtained using canonical *NVT* Metropolis Monte Carlo simulations at a series of temperatures in the range 1-400 K. Further DFT functional tests were performed with PBE [22], BLYP [5, 6] and B3LYP [5, 6, 23] (*vide infra*).

Chemical shifts are calculated with the approximation

$$\delta = \frac{\sigma(\text{Xe atom}) - \sigma(\text{system})}{1 - \sigma(\text{Xe atom})} \approx \sigma(\text{Xe atom}) - \sigma(\text{system})$$

that holds well when the reference shielding constant is small. In this case the error is *ca*. 0.14 ppm in the cage cavity.

3.3 Computed chemical shifts at the centers of the cavities

The calculated xenon NMR shielding constants and chemical shifts with respect to atomic Xe computed with ADF code using jcpl/TZP basis sets are listed in Table S5. Not accounting for dynamical contributions, the best computational static (Stat) estimates for the chemical shifts of Xe wrt. a free Xe atom (at SO-ZORA/BHandHLYP level for Xe atom at the center of the corresponding cavity) is *ca*. -21.1 ppm in the cage and +181.4 ppm in the window cavity. Hence, Xe is shielded at the centre of the cage cavity (negative chemical shift wrt. a free Xe atom, corresponding to a low-pressure Xe gas reference), unlike the typical deshielding that it experiences in the window cavity. Xe atom in the cage is therefore 202.5 ppm more shielded than inside the window cavity. This shielding difference is 242.0, 247.6, and 222.1 ppm with PBE, BLYP, and B3LYP functionals, respectively, at the same theoretical SO-ZORA level.

Table S5. Calculated ^{129}Xe shielding constants and chemical shifts with respect to atomic Xe in the cage and window positions using ADF code. Calculations at the non-relativistic (NR), scalar relativistic (SR-ZORA) and scalar and spin-orbit relativistic (SO-ZORA) levels of theory, using jcpl/TZP basis sets for Xe/other atoms.

		Shielding constant (ppm)			CS wrt.Xe-atom (ppm)	
Functional	Level	Xe atom	Cage	Window	Cage	Window
PBE	NR	5643.2	5649.4	5445.9	-6.2	197.3
	SR-ZORA	5752.2	5755.7	5523.6	-3.6	228.6
	SO-ZORA	6598.0	6600.1	6358.1	-2.1	239.9
aBLYP	NR	5643.6	5649.0	5439.7	-5.6	203.9
	SR-ZORA	5752.6	5755.5	5517.6	-2.9	235.0
	SO-ZORA	6598.6	6600.1	6352.5	-1.5	246.1
B3LYP	NR	5643.3	5656.6	5467.8	-13.3	175.5
	SR-ZORA	5752.1	5763.0	5548.9	-10.9	203.2
	SO-ZORA	6600.5	6608.9	6386.8	-8.4	213.7
	SO-ZORA, FXC[a]	6607.4	6615.6		-8.2	
BHandHLYP	NR	5643.4	5667.9	5497.6	-24.5	145.8
	SR-ZORA	5752.3	5775.6	5581.1	-23.3	171.2
	SO-ZORA	6604.0	6625.1	6422.6	-21.1	181.4

[a]Improved exchange-correlation kernel in ADF 2016.

3.4 DFT functional tests

Increasing the portion of exact exchange in the DFT functional series BLYP, B3LYP and BHandHLYP result in larger shielding (more negative chemical shifts) but smaller shielding difference between the cage and window cavity. These results are in accordance with earlier findings that the pure DFT functionals tend to overestimate the Xe CS, and while there is some overestimation with BHandHLYP as well, it is typically closer to the correlated *ab initio* methods as compared to BLYP and B3LYP [24−28]. PBE and BLYP yield highly similar, *i.e.*, overestimated results at all levels of theory. Hence, the hybrid BHandHLYP is expected to produce the best results for both chemical shifts, resulting

also in the best estimation for the shielding difference between the cage and window cavities. The hybrid DFT effects can be taken into account via scaling of the periodic GGA results.

3.5 Relativistic contributions to Xe chemical shift

With the BHandHLYP functional, relativistic effects at the SO-ZORA level increase the chemical shifts by *ca.* +3.4 ppm (14%) and +35.6 ppm (24%) in the cage and window cavities, respectively, as shown in Table S5 and Figures S18 and S19. The other hybrid functional, B3LYP, produces qualitatively similar relativistic effects (+4.9 ppm for cage and +38.2 ppm for window). The pure DFT functionals, PBE and BLYP, give slightly larger relativistic contributions in the window cavity (*ca.* +42 ppm), following the general trend of overestimation by them. In the window cavity, the main relativistic contribution is SR, which accounts for slightly more than 70% of the effect with all the tested functionals. In the cage cavity the SO outgrows the SR contribution when the amount of exact exchange increases, and with the BHandHLYP functional the SO contribution is larger than SR by 1.0 ppm, covering 65% of the relativistic effects. With pure DFT functionals, the roles are changed. The SO contribution is relatively large but not of equal size in both cage and window cavities and, hence, it should be included as a correction to the SR-ZORA level in periodic modeling.

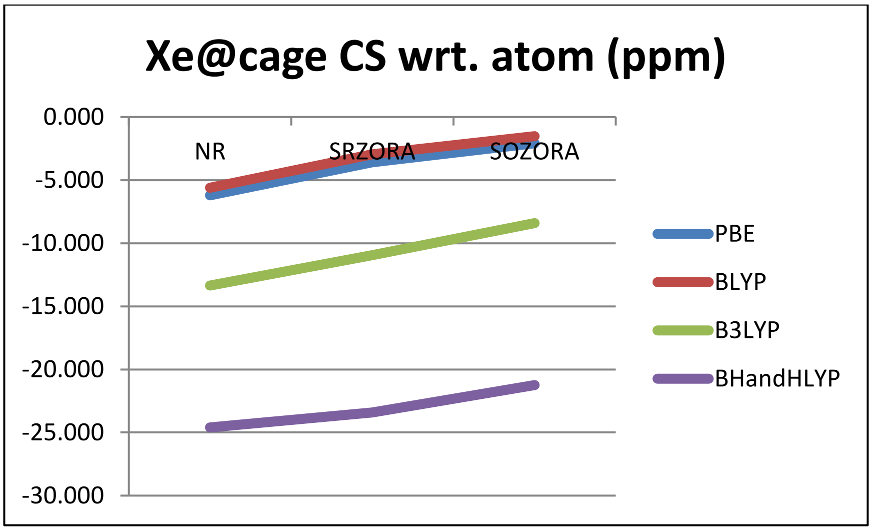

Figure S18. The calculated chemical shifts of Xe in the cage cavity, referenced to atomic Xe, at nonrelativistic (NR), scalar (SR) and spin-orbit coupled (SO) relativistic levels of theory with the pure DFT functionals PBE and BLYP, and the hybrid functionals B3LYP and BHandHLYP.

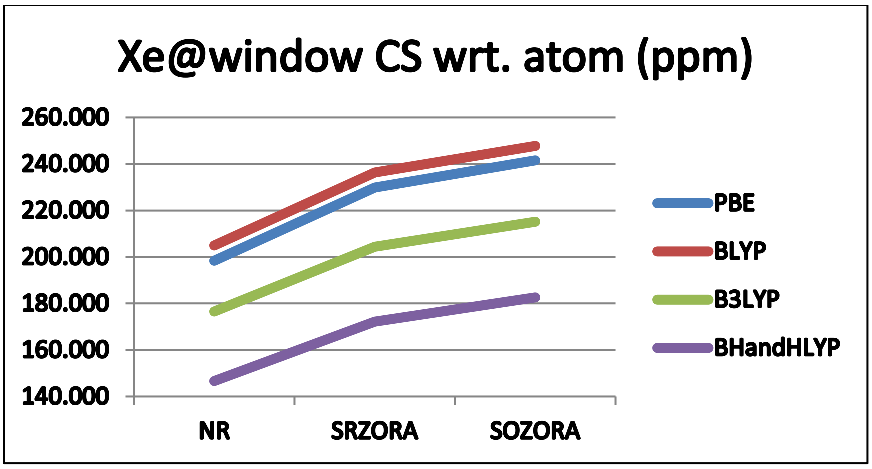

Figure S19. The calculated chemical shifts of Xe in the window cavity, referenced to atomic Xe, at nonrelativistic (NR), scalar (SR) and spin-orbit coupled (SO) relativistic levels of theory with the pure DFT functionals PBE and BLYP, and the hybrid functionals B3LYP and BHandHLYP.

3.6 Exchange-correlation kernel in ADF 2016

The use of the improved exchange-correlation kernel available in ADF version 2016 was tested using the B3LYP functional (see Table S5), and found to account for *ca*. +7 ppm to the isotropic Xe shielding constants of free Xe atom and Xe inside the cage cavity, but resulting in only a negligible, *ca*. +0.2 ppm, modification to the corresponding Xe chemical shift.

3.7 Dynamical modeling and total estimate of the ^{129}Xe chemical shift

Dynamical modeling yielded positive contributions to the ^{129}Xe chemical shift in both cage and window cavities, as shown in Table S6 and Figure S20. In short temperature ranges above 50 K, such as the low and high temperature ranges shown in Figure S20, the dynamical contribution is roughly linear, in the cage (window) cavity approximately +74 ppb/K (+90 ppb/K) and +64 ppb/K (+51 ppb/K), correspondingly. The room-temperature dynamical contributions, +43.4 and +29.7 ppm for cage and window cavities, respectively, switch the cage shift positive and add about 16% to the static Xe chemical shift window cavity value (at relativistic SO-ZORA/BHandHLYP level). The total ^{129}Xe chemical shifts are therefore +22.3 ppm (-21.1 ppm + 43.4 ppm) in the cage and +211.1 ppm (181.4 ppm + 29.7 ppm) in the window when both relativistic and dynamical contributions are considered.

Table S6. Dynamical contribution to the ^{129}Xe chemical shift in the cage and window cavities, referenced to Xe chemical shifts at the center of the corresponding cavity. ^{129}Xe nuclear shieldings were computed at NR level using Turbomole code with BHandHLYP functional and co-r(Xe)/def2-SVP(other) basis sets. The shielding values are 5637.3 ppm for free Xe atom, 5659.7 ppm for Xe at the center of the cage cavity, and 5503.2 ppm for Xe at the center of the window cavity.

Simulation *T* (K)	Xe@cage chemical shift (ppm)	Xe@window chemical shift (ppm)
1	42.7	0.6
5	32.6	2.8
10	28.7	4.4
25	25.5	7.3
50	25.8	10.7
75	27.3	13.5
100	29.0	15.9
125	30.9	18.2
150	32.9	20.2
175	34.6	22.1
200	36.3	23.8
225	38.1	25.5
250	39.5	27.0
275	41.7	28.3
300	43.4	29.7
325	44.8	30.9
350	46.1	32.1
375	47.6	33.1
400	49.3	34.3

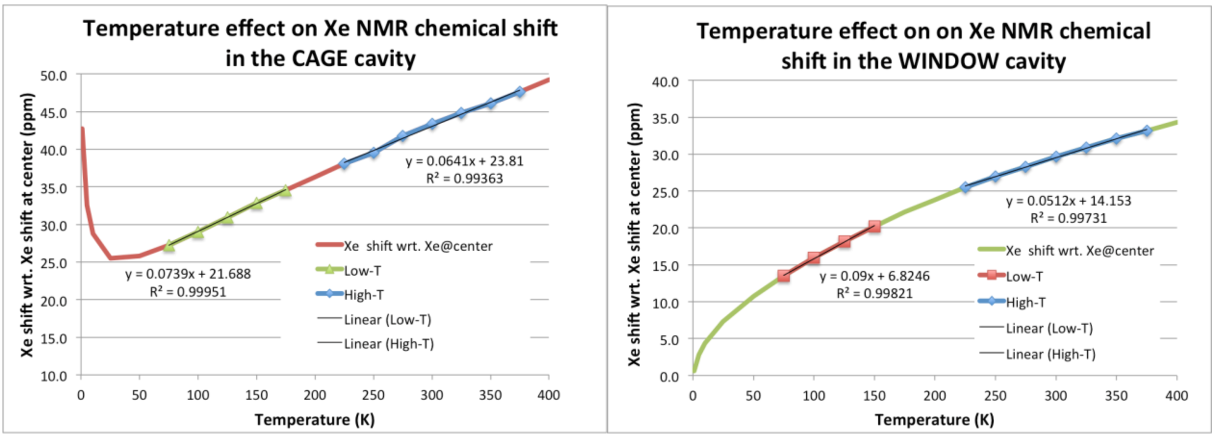

Figure S20. Temperature effect on Xe shift averaged over Xe motion inside cavities.

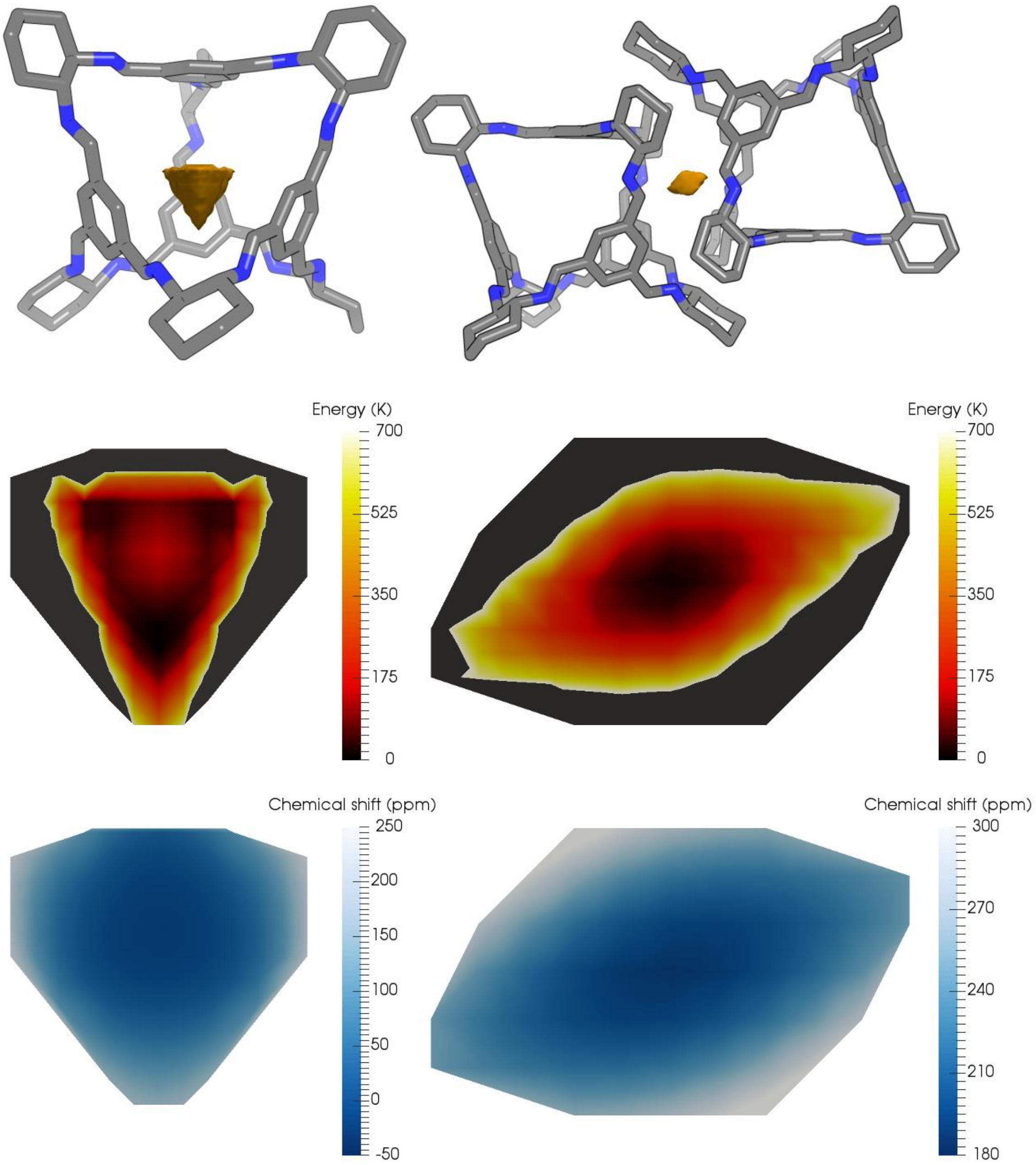

Figure S21. Calculated 3D potential energy and chemical shift surfaces of Xe atom in the cage (left) and window (right) cavities. Top: potential energy isosurfaces at *T* = 350 K. Middle: 2D slice of the potential energy surface up to 700 K (higher values in gray). Bottom: 2D slice of the Xe chemical shift surface. The 2D slices are along the plane containing Xe at the center of the cavity, with the viewpoint given by the corresponding top figure.

3.8 Loading effect on Xe shifts in cage and window cavities

The effect due to different Xe loadings in neighboring cavities is tested with models including one, two, and five cages shown in Figure S17 and the results are listed in Table S7. The full loading of the neighboring cavities decreases the Xe chemical shift, *i.e.* Xe becomes more shielded, as compared to the case where cavities are empty. The effect is much smaller than relativistic and motional effects of Xe. As the effect is about similar size in both cavities, *ca.* -14 ppm, it has an insignificant effect on the chemical shift difference between cavities.

Table S7. Loading effects on the Xe chemical shift in the cage and window cavities due to different occupations in the neighboring cavities. Computed at NR level using Turbomole code with BHandHLYP functional and co-r(Xe)/def2-SVP(other) basis sets. Shielding for the isolated Xe atom reference is 5637.3 ppm at the same level.

	Shielding (ppm)		Chemical shift (ppm)	
	Cage	Window	Cage	Window
One cage model				
1Xe@Cage	5659.7		-22.5	
Two cages model				
1Xe@Window		5503.2		134.8
1Xe@Window/2@Cages	5659.1	5516.6	-21.9	121.4
Five cages model				
1Xe@Center Cage	5660.3		-23.3	121.4
1Xe@Center Cage/4Xe@Windows	5674.8	5508.0	-37.8	129.9

4. References

[1] M. H. Levitt, *Spin dynamics*: basics of nuclear magnetic resonance, 2nd ed., John Wiley & Sons, Chichester, England; Hoboken, NJ, 2008.
[2] J. Roukala, J. Zhu, C. Giri, K. Rissanen, P. Lantto and V.-V. Telkki, *J. Am. Chem. Soc.,* 2015, **137**, 2464.
[3] G. Saielli, A. Bagno, F. Castiglione, R. Simonutti, M. Mauri and A. Mele, *J. Phys. Chem. B,* 2014, **118**, 13963.
[4] S. Standara, P. Kulhánek, R. Marek and M. Straka, *J. Comput. Chem.,* 2013, **34**, 1890.
[5] A. D. Becke, *Phys. Rev. A,* 1988, **38**, 3098.
[6] C. Lee, W. Yang and R. G. Parr, *Phys. Rev. B,* 1988, **37**, 785.
[7] A. D. Becke, *J. Chem. Phys.,* 1993, **98**, 1372.
[8] TURBOMOLE, V6.5; TURBOMOLE GmbH: Karlsruhe, Germany (2013); http://www.turbomole.com.

[9] G. Schreckenbach and T. Ziegler, *J. Phys. Chem.,* 1995, **99**, 606.
[10] ADF2014, SCM, Theoretical Chemistry, Vrije Universiteit, Amsterdam, The Netherlands, http: //www.scm.com.
[11] ADF2016, SCM, Theoretical Chemistry, Vrije Universiteit, Amsterdam, The Netherlands, http: //www.scm.com.
[12] E. van Lenthe, E. J. Baerends and J. G. Snijders, *J. Chem. Phys.,* 1993, **99**, 4597.
[13] E. van Lenthe, E. J. Baerends and J. G. Snijders, *J. Chem. Phys.,* 1994, **101**, 9783.
[14] E. van Lenthe, J. G. Snijders and E. J. Baerends, *J. Chem. Phys.,* 1996, **105**, 6505.
[15] S. K. Wolff and T. Ziegler, *J. Chem. Phys.,* 1998, **109**, 895.
[16] S. K. Wolff, T. Ziegler, E. van Lenthe, and E. J. Baerends, *J. Chem. Phys.,* 1999, **110**, 7689.
[17] J. Autschbach, *ChemPhysChem,* 2009, **10**, 2274.
[18] S. Grimme, J. Anthony, S. Ehrlich and H. Krieg, *J. Chem. Phys.,* 2010, **132**, 154104.
[19] J. Vaara, M. Hanni and J. Jokisaari, *J. Chem. Phys.,* 2013, **138**, 104313.
[20] M. Hanni, P. Lantto M. Repiský, J. Mareš, B. Saam, and J. Vaara, 2016, *submitted for publication*.
[21] (a) A. Schäfer, H. Horn and R. J. Ahlrichs, *Chem. Phys.,* 1992, **97**, 2571. (b) F. Weigend and R. Ahlrichs, *Phys. Chem. Chem. Phys.,* 2005, **7**, 3297.
[22] J. P. Perdew, K. Burke and M. Ernzerhof, *Phys. Rev. Lett.,* 1996, **77**, 3865. Erratum Ibid. **78**, 1396 (1997).
[23] A. D. Becke, *J. Chem. Phys.,* 1993, **98**, 5648.
[24] P. Lantto and J. Vaara, *J. Chem. Phys.,* 2007, **127**, 084312.
[25] M. Straka, P. Lantto, M. Räsänen and J. Vaara, *J. Chem. Phys.,* 2007, **127**, 234314.
[26] M. Straka, P. Lantto and J. Vaara, *J. Phys. Chem. A,* 2008, **112**, 2658.
[27] A. M. Kantola, P. Lantto, J. Vaara and J. Jokisaari, *Phys. Chem. Chem. Phys.,* 2010, **12**, 2679.
[28] J. Roukala, A. F. Maldonado, J. Vaara, G. A. Aucar and P. Lantto, *Phys. Chem. Chem. Phys.,* 2011, **13**, 21016.
